# Heterogeneous expression pattern of interleukin 17A (IL-17A), IL-17F and their receptors in synovium of rheumatoid arthritis, psoriatic arthritis and osteoarthritis: possible explanation for nonresponse to anti-IL-17 therapy?

**DOI:** 10.1186/s13075-014-0426-z

**Published:** 2014-08-22

**Authors:** Lisa GM van Baarsen, Maria C Lebre, Dennis van der Coelen, Saïda Aarrass, Man W Tang, Tamara H Ramwadhdoebe, Daniëlle M Gerlag, Paul P Tak

**Affiliations:** Division of Clinical Immunology and Rheumatology, Academic Medical Centre, University of Amsterdam, Meibergdreef 9, 1105, AZ Amsterdam, the Netherlands; Department of Experimental Immunology, Academic Medical Centre, University of Amsterdam, Meibergdreef 9, 1105 AZ, Amsterdam, The Netherlands; Current address: University of Cambridge, U.K. Trinity Lane, Cambridge CB2 1TN, UK, and GlaxoSmithKline, Stevenage UK Gunnels Wood Road, Stevenage Herts SG1 2NY, UK

## Abstract

**Introduction:**

Accumulating evidence suggests an important role for interleukin 17 (IL-17) in the pathogenesis of several inflammatory diseases, including rheumatoid arthritis (RA) and psoriatic arthritis (PsA). Accordingly, clinical trials aimed at blocking IL-17 have been initiated, but clinical results between patients and across different diseases have been highly variable. The objective was to determine the variability in expression of IL-17A, IL-17F and their receptors IL-17RA and IL-17RC in the synovia of patients with arthritis.

**Methods:**

Synovial biopsies were obtained from patients with RA (*n* = 11), PsA (*n* = 15) and inflammatory osteoarthritis (OA, *n* = 14). For comparison, synovia from noninflamed knee joints (*n* = 7) obtained from controls were included. Frozen sections were stained for IL-17A, IL-17F, IL-17RA and IL-17RC and evaluated by digital image analysis. We used confocal microscopy to determine which cells in the synovium express IL-17A and IL-17F, double-staining with CD4, CD8, CD15, CD68, CD163, CD31, von Willebrand factor, peripheral lymph node address in, lymphatic vessel endothelial hyaluronan receptor 1, mast cell tryptase and retinoic acid receptor–related orphan receptor γt (RORγt).

**Results:**

IL-17A, IL-17F, IL-17RA and IL-17RC were abundantly expressed in synovial tissues of all patient groups. Whereas IL-17RA was present mostly in the synovial sublining, IL-17RC was abundantly expressed in the intimal lining layer. Digital image analysis showed a significant (*P* < 0.05) increase of only IL-17A in arthritis patients compared to noninflamed control tissues. The expression of IL-17A, IL-17F and their receptors was similar in the different patient groups, but highly variable between individual patients. CD4+ and CD8+ cells coexpressed IL-17A, and few cells coexpressed IL-17F. IL-17A and IL-17F were not expressed by CD15+ neutrophils. Mast cells were only occasionally positive for IL-17A or IL-17F. Interestingly, IL-17A and IL-17F staining was also observed in macrophages, as well as in blood vessels and lymphatics. This staining probably reflects receptor-bound cytokine staining. Many infiltrated cells were positive for the transcription factor RORγt. Colocalisation between RORγt and IL-17A and IL-17F indicates local IL-17 production.

**Conclusions:**

Increased expression of IL-17A is not restricted to synovial tissues of RA and PsA patients; it is also observed in inflammatory OA. The heterogeneous expression levels may explain nonresponse to anti-IL-17 therapy in subsets of patients.

**Electronic supplementary material:**

The online version of this article (doi:10.1186/s13075-014-0426-z) contains supplementary material, which is available to authorized users.

## Introduction

Whereas initial studies focused mainly on type 1 helper T (Th1) cells as having a prominent role in rheumatoid arthritis (RA), subsequent studies suggested that Th17 cells also have an important role in this disease. The functional role of this new type of effector helper T cell has been studied extensively in recent years and shows that it has important functions in the response to microbial infection and in promoting and maintaining chronic inflammation and autoimmunity [1]. Interestingly, recent studies have demonstrated the plasticity of Th17 cells and have indicated their pathogenic role, especially when shifted towards Th1 cells [2-4].

Th17 cells were originally identified by their expression of the proinflammatory cytokine interleukin 17 (IL-17) and represent a distinct subset of CD4+ cells characterised by the expression of Retinoic acid receptor–related orphan receptor γt (RORγt) [5]. The production of IL-17 is specifically regulated by this transcription factor. The cytokine IL-17 is formally referred to as IL-17A and is part of a larger IL-17 cytokine family consisting of six members ranging from IL-17A to IL-17F. The most studied family members are IL-17A and IL-17F, which are mainly produced by Th17 cells to protect against extracellular pathogens and fungi but also have proinflammatory properties. IL-17 promotes the transcriptional activation of proinflammatory cytokines, hematopoietic cytokines, acute-phase response genes and antimicrobial mediators [6], especially in synergy with other proinflammatory cytokines such as tumour necrosis factor and IL-1β [7,8]. The common receptor for the IL-17 Family members is IL-17RA [9]. In addition to IL-17RA, IL-17RC is required for signalling in response to IL-17A or IL-17F [10,11].

Levels of IL-17 have been found to be increased in the synovial fluid and tissues of RA patients [12-14]. Furthermore, studies in several experimental animal models demonstrated a detrimental role of IL-17 in arthritis [15-17]. IL-17^−/−^ mice are resistant to collagen-induced arthritis [18]. Therefore, it has been suggested that IL-17 may play a crucial role in the pathogenesis of different forms of arthritis by inducing synovial inflammation and promoting bone destruction [19,20]. Accordingly, several clinical trials targeting the IL-17 pathway in RA [21], psoriasis [22,23], psoriatic arthritis (PsA) [24] and ankylosing spondylitis [25] have been initiated (reviewed in [26]). Although psoriasis patients show marked clinical benefit of targeting IL-17A, the clinical response in RA and PsA has been limited and variable between patients [27]. Conceivably, inhibiting IL-17 is only beneficial for those arthritis patients with increased cytokine levels in the target tissue. To provide more insight into the heterogeneity of the expression of IL-17 and IL-17 receptors at the site of inflammation, we investigated the expression of IL-17A, IL-17F and their receptors at the protein level in synovial tissues from inflammatory arthritis patients.

## Methods

### Patients and synovial tissue collection

We included biological naïve patients with RA (according to the American College of Rheumatology (ACR) 1987 criteria [28], *n* = 11), PsA (Caspar criteria [29], *n* = 15) and inflammatory osteoarthritis (OA; according to the 1986 ACR criteria for OA [30], *n* = 14). For research purposes all patients underwent miniarthroscopic synovial biopsy sampling of an actively inflamed ankle or knee joint [31]. Six to eight samples were collected for immunohistochemistry (IHC) to correct for sampling error, as described previously [32]. For comparison non-inflammatory controls (n = 7) were included, patients who underwent orthopaedic arthroscopy for non-inflammatory joint pain. Table 1 shows the demographic and clinical features. The study was approved by the Institutional Review Board of the Academic Medical Centre and performed according to the Declaration of Helsinki. All study patients provided written informed consent.

**Table 1:** **Demographic and clinical features** ^**a**^

	**Controls (** ***n*** **= 7)**	**RA (** ***n*** **= 11)**	**PsA (** ***n*** **= 15)**	**OA (** ***n*** **= 14)**
Age, yr	47 (40 to 60)	49 (45 to 62)	48 (34 to 56)	68 (54 to 77)
Females, *n* (%)	2 (29)	7 (64)	7 (47)	10 (71)
Disease duration, mo	NA	18 (8 to 88)	41 (23 to 84)^b^	42 (13 to 72)^c^
Erosive disease, *n* (%)	NA	3/8 (38)	ND	ND
RF-positive, *n*/total (%)	NA	3/11 (27)	ND	ND
ACPA-positive, *n* (%)	NA	4/7 (57)	ND	ND
VAS GDA	NA	57 (55 to 85)	67 (54 to 75)	ND
TJC28, *n*	NA	9 (7 to 13)	2 (1 to 12)	ND
SJC28	NA	11 (6 to 14)	2 (1 to 12)	ND
ESR, mm/h	NA	23 (16 to 47)	34 (8 to 56)	ND
CRP, mg/L	NA	24.3 (12.0 to 37.0)	11.0 (4.7 to 18.0)	ND
DAS28	NA	5.46 (5.33 to 6.62)	4.67 (2.83 to 5.99)	ND
NSAID use, *n* (%)	NA	7 (64)	6 (40)	6 (67)^d^
MTX use, *n* (%)	NA	6 (55)	10 (67)	0 (0)

^a^ACPA, Anticitrullinated protein antibodies; CRP, C-reactive protein; DAS28, Disease Activity Score in 28 joints; ESR, Erythrocyte sedimentation rate; MTX, Methotrexate; NA, Not applicable; ND, Not determined; NSAID, Nonsteroidal anti-inflammatory drug; OA, Osteoarthritis; PsA, Psoriatic arthritis; RA, Rheumatoid arthritis; RF, Rheumatoid factor; SJC28, Swollen joint count of 28 joints; TJC28, Tender joint count of 28 joints; VAS GDA, Visual analogue scale (range from 0 to 100 mm) global disease activity. ^b^ *n* = 1, ^c^ *n* = 6, ^d^ *n* = 5 with no data available. Data are presented as median (interquartile range).

To ensure preservation of antigenic content [31,32], synovial biopsy samples were snap-frozen *en bloc* in Tissue-Tek O.C.T. compound (Sakura Finetek Europe, Zoeterwoude, the Netherlands) immediately after collection and stored in liquid nitrogen. Synovial tissue biopsies were cut into 5-μm sections and mounted on StarFrost adhesive glass slides (Knittelgläser, Braunschweig, Germany), after which slides were sealed in parafilm (Bemis, Neenah, WI, USA) and stored at −80°C until further use.

### Antibodies

To investigate the detailed expression pattern of IL-17 in synovium, tissue sections were stained using mouse monoclonal antibodies against IL-17A (immunoglobulin G1 (IgG1), clone 41802), IL-17F (IgG2a, clone 197315) and their receptors IL-17RA (IgG1, clone 133617) and IL-17RC (IgG2b, clone 309882), all from R&D Systems (Minneapolis, MN, USA). For colocalisation studies, we used antibodies against the transcription factor RORγt (RORc, mouse IgG2a; RayBiotech, Norcross, GA, USA), T cells (biotin-conjugated mouse anti-CD4: IgG1, clone RPA-T4; Biolegend, San Diego, CA, USA; and biotin-conjugated mouse anti-CD8: IgG1, clone RFT8; SouthernBiotech, Birmingham, AL), B cells (biotin-conjugated mouse anti-CD19; IgG1, clone HIB19; Biolegend), macrophages (biotin-conjugated mouse anti-CD68; IgG2b clone Y1/82A; Biolegend; and biotin-conjugated mouse anti-CD163; IgG1 clone GHI/61; Biolegend), neutrophils (biotin-conjugated mouse anti-CD15; IgM clone HI98; eBioscience, San Diego, CA, USA), mast cells (mouse anti–mast cell tryptase (MCT); IgG1 clone AA1; Dako, Glostrup, Denmark), lymphatic vessels (goat polyclonal anti-lymphatic vessel endothelial hyaluronan receptor 1 (Lyve-1); R&D Systems), blood vessels (mouse anti–von Willebrand factor (vWF); IgG1 clone F8/86; Dako) and high endothelial venules (mouse anti–peripheral lymph node addressin (PNAd); IgM clone MECA-79; Biolegend). Because some cell-specific antibodies were of the same isotype as the IL-17A antibody, we used a rabbit polyclonal IL-17A antibody (Insight Biotechnology, Wembley, UK) to investigate colocalisation with MCT, CD31 and vWF. During this project, the anti-IL-17F antibody was removed from the market and replaced with another mouse monoclonal anti-IL-17F antibody (IgG2b clone 197301; R&D Systems). Colocalisation studies using both anti-IL-17F antibodies showed that the antibodies almost completely overlap.

### Immunohistochemistry

IHC was performed using a two-step immunoperoxidase method followed by a biotin tyramide (PerkinElmer, Waltham, MA, USA) enhancement step to detect IL-17A, IL-17F and IL-17RA or followed by BrightVision (Immunologic, Duiven, the Netherlands) to detect IL-17RC. Sealed slides containing frozen sections were thawed at room temperature for 30 minutes, unpacked, and air-dried for another 20 minutes. Subsequently, sections were fixed in acetone, and endogenous peroxidase activity was blocked with 0.3% H_2_O_2_ in 0.1% sodium azide in phosphate-buffered saline (PBS) for 20 minutes. After washing in PBS, primary antibodies were incubated overnight at 4°C. As negative controls, irrelevant isotype-matched immunoglobulins instead of the primary antibody were applied to the sections. The next day, staining was developed using a goat anti-mouse horseradish peroxidase (HRP)–conjugated antibody (Dako), after which the intensity was enhanced using biotinylated tyramide (PerkinElmer) and streptavidin-HRP (Dako). Enhancement of the IL-17RC staining was performed using BrightVision. 3-Amino-9-ethylcarbazole; Vector Laboratories, Burlingame, CA, USA) was used as chromogen. Slides were counterstained with Gill’s haematoxylin (Klinipath, Duiven, the Netherlands) and mounted in Kaiser’s glycerol gelatin (Merck, Darmstadt, Germany).

The intensity of staining was analysed by digital image analysis in a blinded fashion using a Syndia algorithm on a Qwin-based analysis system (Leica, Cambridge, UK) as described previously [33]. The expression of stained proteins was calculated for each section as the median integrated optical density per square millimetre of tissue [34].

### Colocalisation using immunofluorescence

Colocalisation studies between IL-17 and cell-specific markers were performed using a sequential double-immunofluorescence staining method. Frozen tissue sections were thawed and air-dried at room temperature and subsequently fixed in acetone. After washing in PBS, primary antibody (anti-IL17A or anti-IL-17F) was applied and incubated overnight at 4°C. As a negative control, sections were incubated with isotype-matched immunoglobulins. After incubation, bound primary antibodies were detected using isotype-specific Alexa Fluor 594–conjugated or Alexa Fluor 488–conjugated antibodies (Molecular Probes Europe, Leiden, the Netherlands). Subsequently, sections were blocked with 10% normal mouse serum to prevent nonspecific binding. Cell-specific antibodies were applied and incubated for 1 hour at room temperature, after which the staining was developed using streptavidin-Alexa Fluor 594–, streptavidin-Alexa Fluor 633– or isotype-specific Alexa Fluor 594– or Alexa Fluor 488–conjugated antibodies (Molecular Probes Europe). After staining, the slides were mounted with VECTASHIELD HardSet containing 4′,6-diamidino-2-phenylindole dihydrochloride (Vector Laboratories) for nuclear counterstaining. Colocalisation was visualised using a TCS SP2 spectral confocal and multiphoton system (Leica Microsystems, Wetzlar, Germany).

### Statistical analysis

GraphPad Prism software (V.5; GraphPad Software, La Jolla, CA, USA) and SPSS version 18.0.2 software (SPSS, Chicago, IL, USA) were used for statistical analysis. Nonnormally distributed data are presented as median (interquartile range). Differences between study groups were analysed using the Kruskal-Wallis test with *post hoc* Dunn’s multiple comparisons test or the Mann Whitney *U* test where appropriate. Correlations were assessed with the Spearman’s rank-order correlation coefficients. *P*-values below 0.05 were considered statistically significant.

## Results

### Levels of IL-17A were significantly increased in synovial tissues of patients with inflammatory arthritis, but highly variable between patients

**Figure 1:**
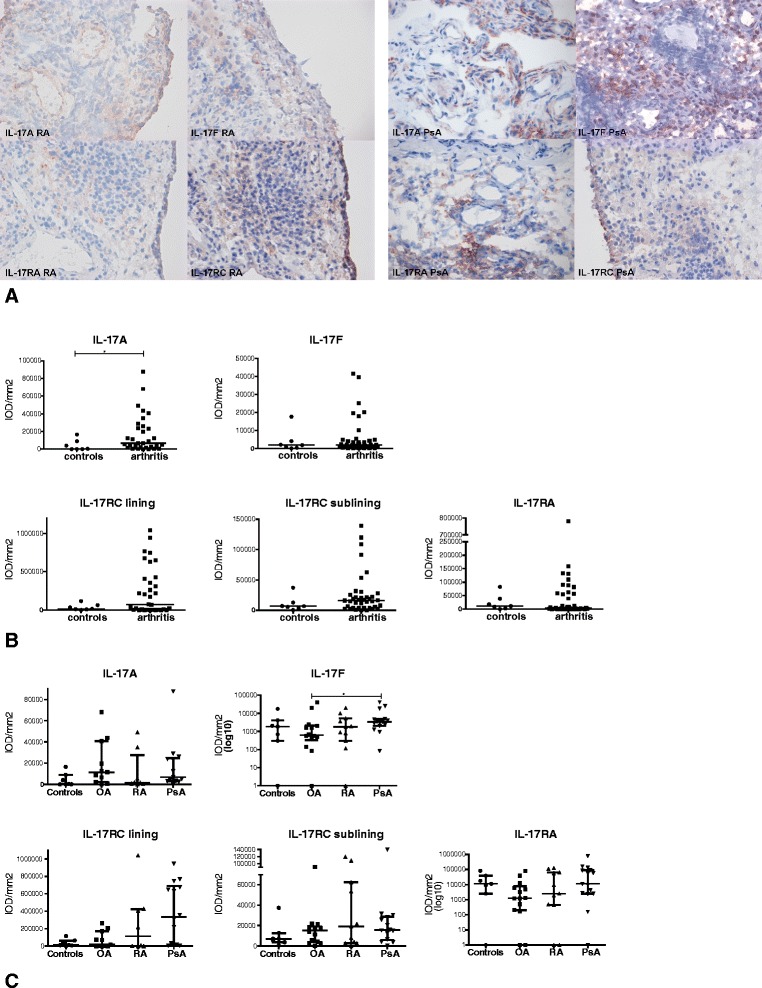
**Expression of interleukins 17A and 17F and their receptors in synovial tissue.** The expression of interleukin 17A (IL-17A), IL-17F, IL-17RA and IL-17RC in rheumatoid arthritis (RA), osteoarthritis (OA), psoriatic arthritis (PsA) and noninflamed synovial tissue was determined by immunohistochemistry using monoclonal antibodies. For each marker, a representative microscopic picture is given in synovial tissue of a RA patient (left) and a PsA patient (right) **(A)**. The intensity of staining was analysed using digital image analysis, and levels were compared between the noninflammatory and arthritis groups using a Mann-Whitney *U* test **(B)** and between OA, PsA and RA patients using a Kruskal-Wallis test with *post hoc* Dunn’s multiple-comparisons tests **(C)**. *P < 0.05. IOD, Integrated optical density.

We used monoclonal antibodies against IL-17A, IL-17F, IL-17RA and IL-17RC to determine their protein expression in synovial tissue biopsies using IHC (Figure 1A). A diffuse cytoplasmic staining pattern was observed for IL-17A in the sublining as well as in the intimal lining layer of the synovium. IL-17F showed a more cellular staining pattern, mainly in the synovial sublining. In line with their ubiquitous expression, both IL-17RA and IL-17RC were abundantly present at high IL-17RC levels in the intimal lining layer. Whereas the levels of IL-17F, IL-17RA and IL-17RC were on average comparable between noninflamed synovial tissues (*n* = 7) and the levels observed in patients with inflammatory arthritis (*n* = 40), the levels of IL-17A were significantly increased in patients with inflammatory arthritis (Figure 1B).

The expression levels of IL-17A and their receptors were not different between RA, OA and PsA patients (Figure 1C). This is in line with the results of the study by Benham and colleagues, which showed no difference in synovial IL-17 expression between RA, PsA and OA patients, although those investigators did not (further) stain for the different members of the IL-17 Family and their receptors [35]. The levels of IL-17F were significantly increased in PsA compared to OA patients.

Interestingly, the overall levels of IL-17RA were positively correlated with IL-17F (*r* = 0.45, *P* = 0.001), but not with IL-17A (*r* = 0.15, *P* = 0.36). In addition, the overall IL-17RC level was positively correlated with both IL-17A and IL-17F (*r* = 0.48, *P* = 0.005; and *r* = 0.39, *P* = 0.012, respectively). In a subanalysis, similar correlations were observed for individual patients within a group of the same diagnosis (data not shown). This may suggest that targeting IL-17RC might be a more effective treatment strategy through inhibition of both the IL-17A and IL-17F pathways.

The baseline characteristics between the different groups of patients were comparable, except for age and swollen joint count 28 (SJC28). OA patients were significantly older than the other groups. The RA patients had more swollen joints compared to the PsA patients. The expression levels of IL-17A, IL-17F, IL-17RA and IL-17RC were not correlated with clinical parameters. However, IL-17RA was positively correlated with the erythrocyte sedimentation rate (ESR) and C-reactive protein (*r* = 0.41 and *P* = 0.040, *r* = 0.41 and *P* = 0.045, respectively). The correlation with ESR was strongest in the PsA patients (*r* = 0.50 and *P* = 0.028). Only the levels of IL-17F showed a weak but statistically significant negative correlation with age (*r* = −0.41, *P* < 0.005). We cannot exclude the possibility that this may be explained by chance in light of multiple comparisons, although the *P*-value was quite low.

Altogether, these immunohistochemical analyses revealed a striking heterogeneous expression pattern for IL-17A, IL-17F and their receptors in inflamed synovium. Although some synovial tissues showed high expression levels, in other patients almost no expression was observed. This variability may explain the variable clinical responses observed in clinical trials in which IL-17 was targeted (reviewed in [27,36]). In addition, low levels of IL-17 may reflect the possible shift from Th17 to Th1 cells at sites of local inflammation [4,37].

### Cellular sources of IL-17A and IL-17F in inflamed synovium

Conflicting data have been reported on the cellular sources of IL-17A and IL-17F in inflamed synovia of patients with different forms of arthritis. In the first studies showing IL-17 production and protein expression in RA synovial tissue biopsies using IHC, researchers reported lymphocytic morphology of IL-17-positive cells [12], and IL-17A and IL-17F staining was especially prominent in lymphocyte aggregates [12,38-41]. IL-17A-producing CD4+ T cells have also been observed in the synovial fluid of RA patients [42,43] and PsA patients [35,41,44]. Later reports suggested colocalisation of IL-17 with markers for mast cells [45,46] and neutrophils [47] in RA synovial tissue. Also, in ankylosing spondylitis, cells of the innate immune system appeared to be a major source of IL-17 [48,49]. The conflicting observations may be the result of technical difficulties in detecting IL-17 in paraffin-embedded synovial tissue, the use of polyclonal antibodies to detect IL-17, and clinical differences between patients studied.

**Figure 2:**
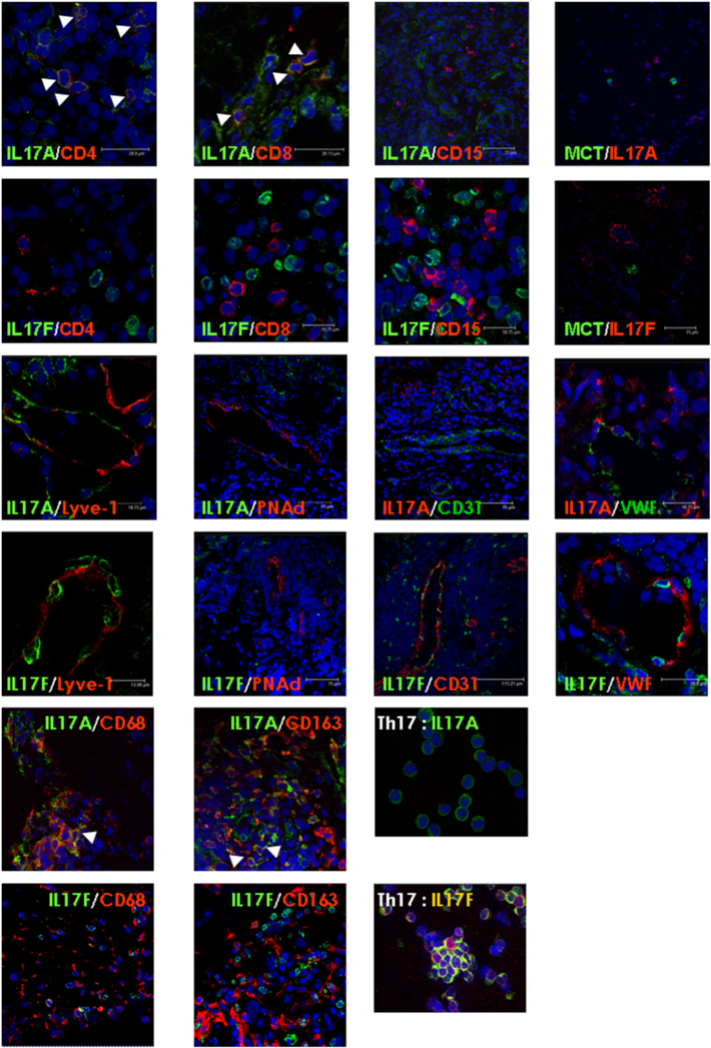
**Colocalisation between interleukins 17A, 17F and cell-specific markers.** The colocalisation between interleukin 17A (IL-17A) or IL-17F and markers for T cells, neutrophils, mast cells, macrophages, blood vessels and lymphatics was examined using double-immunofluorescence labelling and visualised by confocal microscopy. White arrowheads indicate colocalisation. Representative pictures are presented. Type 17 helper T (Th17) cells were used to show that the IL-17A and IL-17F antibodies used can detect Th17-produced IL-17. Lyve-1, Lymphatic vessel endothelial hyaluronan receptor 1; MCT, Mast cell tryptase; PNAd, Peripheral lymph node addressin; vWF, von Willebrand factor. Pictures illustrating the separate channels for CD4, CD8, CD68 and CD163 staining with IL-17 are shown in Additional file 1: Figure S1 and Additional file 2: Figure S2.

We performed detailed double-immunofluorescence studies to investigate colocalisation of IL-17A or IL-17F and markers for T cells, neutrophils, mast cells and macrophages. In addition, because our IHC results indicated the presence of IL-17A and IL-17F in vessel-like structures, we included markers for blood vessels and lymphatics. To investigate whether the origin of IL-17A or IL-17F was different between RA, PsA and OA patients, synovial tissue was stained from at least five different patients per diagnosis for the analysis of T cells, mast cells and macrophages. For the other markers, we stained synovial tissues from at least two different patients per diagnosis. Representative confocal pictures are shown in Figure 2, Additional file 1: Figure S1 and Additional file 2: Figure S2.

The frequency of CD15+ neutrophils in the synovium was very low, and no colocalisation with IL-17A or IL-17F was observed, suggesting that, in inflamed synovium, these cells do not contribute to IL-17 production. CD4+ and CD8+ T cells stained positive for IL-17A, and few cells were positive for IL-17F. CD68+ and CD163+ macrophages showed colocalisation with IL-17A and, to a lesser extent, with IL-17F. We only occasionally observed colocalisation between MCT + mast cells and IL-17A or IL-17F, mainly in PsA patients. Interestingly, we observed IL-17A- and IL-17F-positive cells in vWF+, CD31+ and PNad + blood vessels, as well as in Lyve-1 positive lymphatics. Overall, no clear differences were observed between RA, PsA and OA patients.

**Figure 3:**
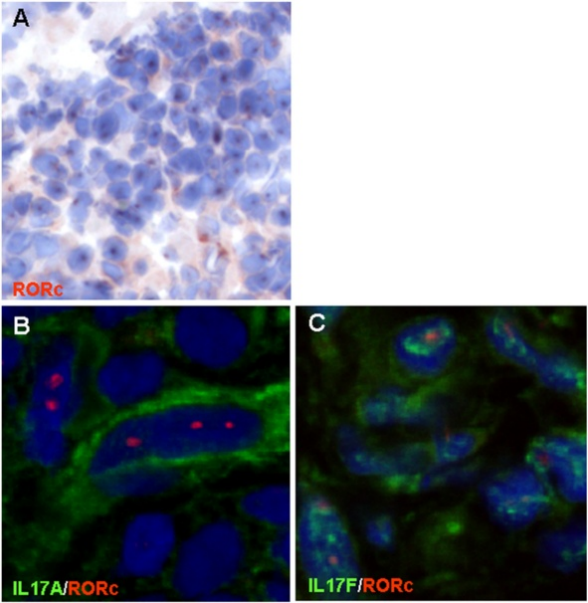
**Retinoic acid receptor–related orphan receptor γt**–**positive cells in synovium.** Immunohistochemistry showed an abundant nuclear expression of the IL-17-specific transcription factor retinoic acid receptor–related orphan receptor γt (RORγt) (shown in red) in synovial tissue biopsies **(A)**. Immunofluorescence showed that RORγt-positive cells (red nuclei) in synovial tissue were indeed positive for interleukin 17A (IL-17A) **(B)** or IL-17F **(C)**.

Colocalisation between IL-17 and specific cell markers does not necessarily mean that IL-17 is produced by this specific cell. Because the used IL-17 antibodies are nonneutralising, we may detect IL-17 bound to its receptor, which probably is the case in the IL-17-positive blood vessels and lymphatics. To investigate whether IL-17 can be produced by infiltrated cells in the synovium, we stained for the transcription factor RORγt, which is essential for IL-17 production. RORγt-positive cells were abundantly present throughout the synovial tissues (Figure 3A). As expected, most RORγt-positive cells were CD3+ T cells (data not shown). More importantly, RORγt nuclear staining colocalised with IL-17A and IL-17F around the same cell, indicating local cytokine production (Figures 3B and C).

## Discussion

We observed increased IL-17A expression in synovial tissues from inflammatory arthritis patients compared with noninflamed control synovium, supporting the concept that IL-17 is a key cytokine in chronic inflammatory arthritis. The levels of IL-17A, IL-17F and their receptors are comparable, on average, between RA, PsA and OA. Using double-immunofluorescence we show that T cells and macrophages can contribute to the production of IL-17A and IL-17F. Colocalisation between the transcription factor RORγt and IL-17 indicate local cytokine production. The heterogeneous expression patterns of IL-17A, IL-17F and their receptors could perhaps explain the limited clinical response observed in clinical trials in which IL-17A in arthritis is targeted, as many patients exhibit very low IL-17 levels at the site of inflammation. These results suggest that patient stratification based on IL-17 expression may be required to demonstrate the beneficial effect of anti-IL-17 therapy in subsets of patients.

## Additional files

Additional file 1: Figure S1. Colocalisation between IL-17A and CD4, CD8, CD68 and CD163 was examined using double-immunofluorescence labelling and visualized by confocal microscopy. Representative pictures showing separate channels are presented. Additional file 2: Figure S2. Colocalisation between IL-17F and CD4, CD8, CD68 and CD163 was examined using double-immunofluorescence labelling and visualized by confocal microscopy. Representative pictures showing separate channels are presented.

## Abbreviations

ACPA: Anticitrullinated protein antibody
CD: Cluster of differentiation
CRP: C-reactive protein
ESR: Erythrocyte sedimentation rate
HRP: Horseradish peroxidase
IHC: Immunohistochemistry
IL: Interleukin
Lyve-1: Lymphatic vessel endothelial hyaluronan receptor 1
MCT: Mast cell tryptase
MTX: Methotrexate
OA: Osteoarthritis
PBS: Phosphate-buffered saline
PNAd: Peripheral lymph node addressin
PsA: Psoriatic arthritis
RA: Rheumatoid arthritis
RF: Rheumatoid factor
RORc: Retinoic acid receptor–related orphan receptor C
RORγ: Retinoic acid receptor–related orphan receptor γ
SJC28: Swollen joint count of 28 joints
TJC28: Tender joint count of 28 joints
VAS GDA: Visual analogue scale (range 0 to 100 mm) of global disease activity
VWF: von Willebrand factor
