# Supplementary figures and images for: Heterogeneous expression pattern of interleukin 17A (IL-17A), IL-17F and their receptors in synovium of rheumatoid arthritis, psoriatic arthritis and osteoarthritis: possible explanation for nonresponse to anti-IL-17 therapy?
Source: Arthritis Res Ther. 2014 Aug 22;16(4):426. doi: 10.1186/s13075-014-0426-z (PMC4292832; doi:10.1186/s13075-014-0426-z)

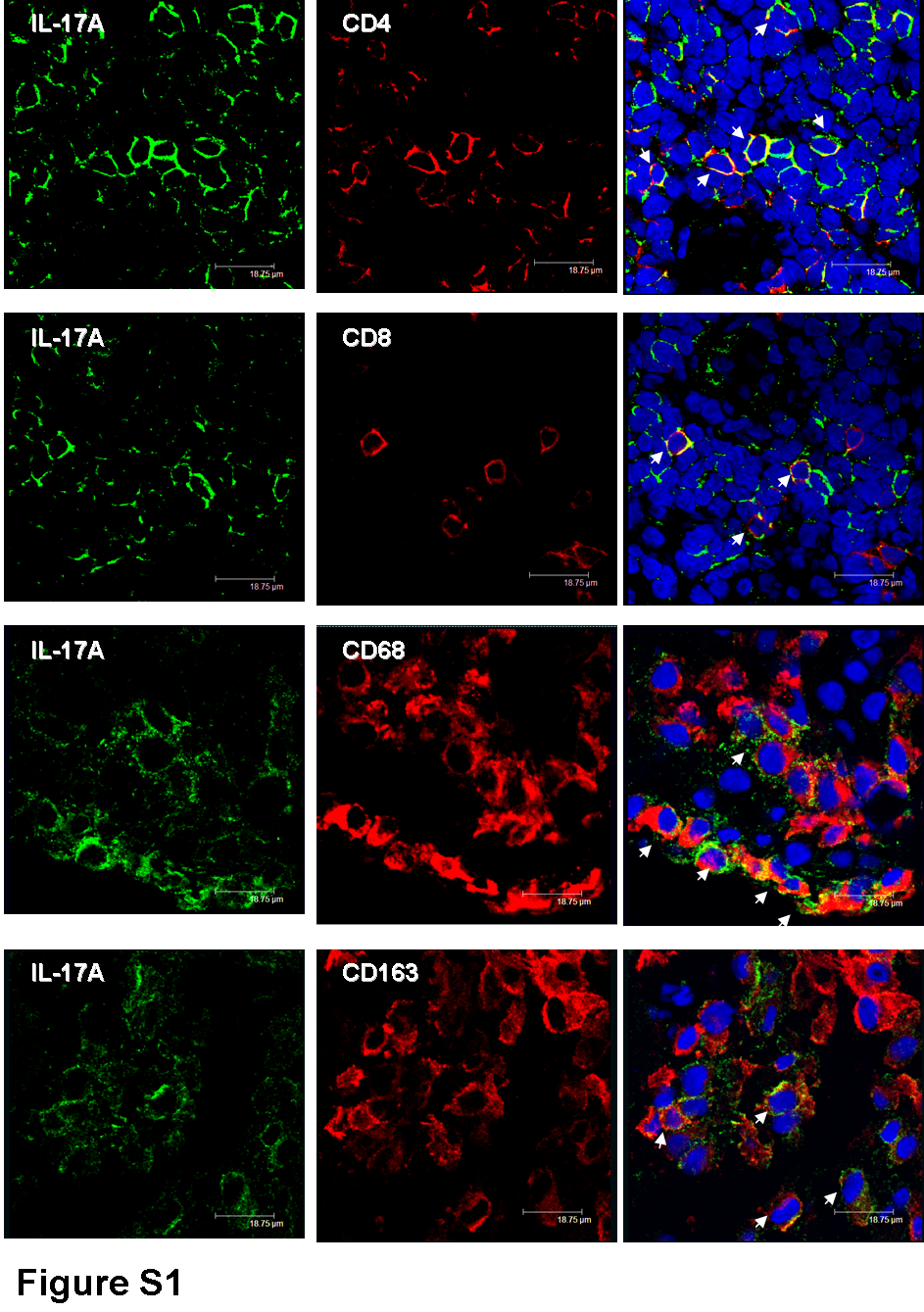

Supplement: Additional file 1: Figure S1. — Colocalisation between IL-17A and CD4, CD8, CD68 and CD163 was examined using double-immunofluorescence labelling and visualized by confocal microscopy. Representative pictures showing separate channels are presented. [file 13075_2014_426_MOESM1_ESM.png]

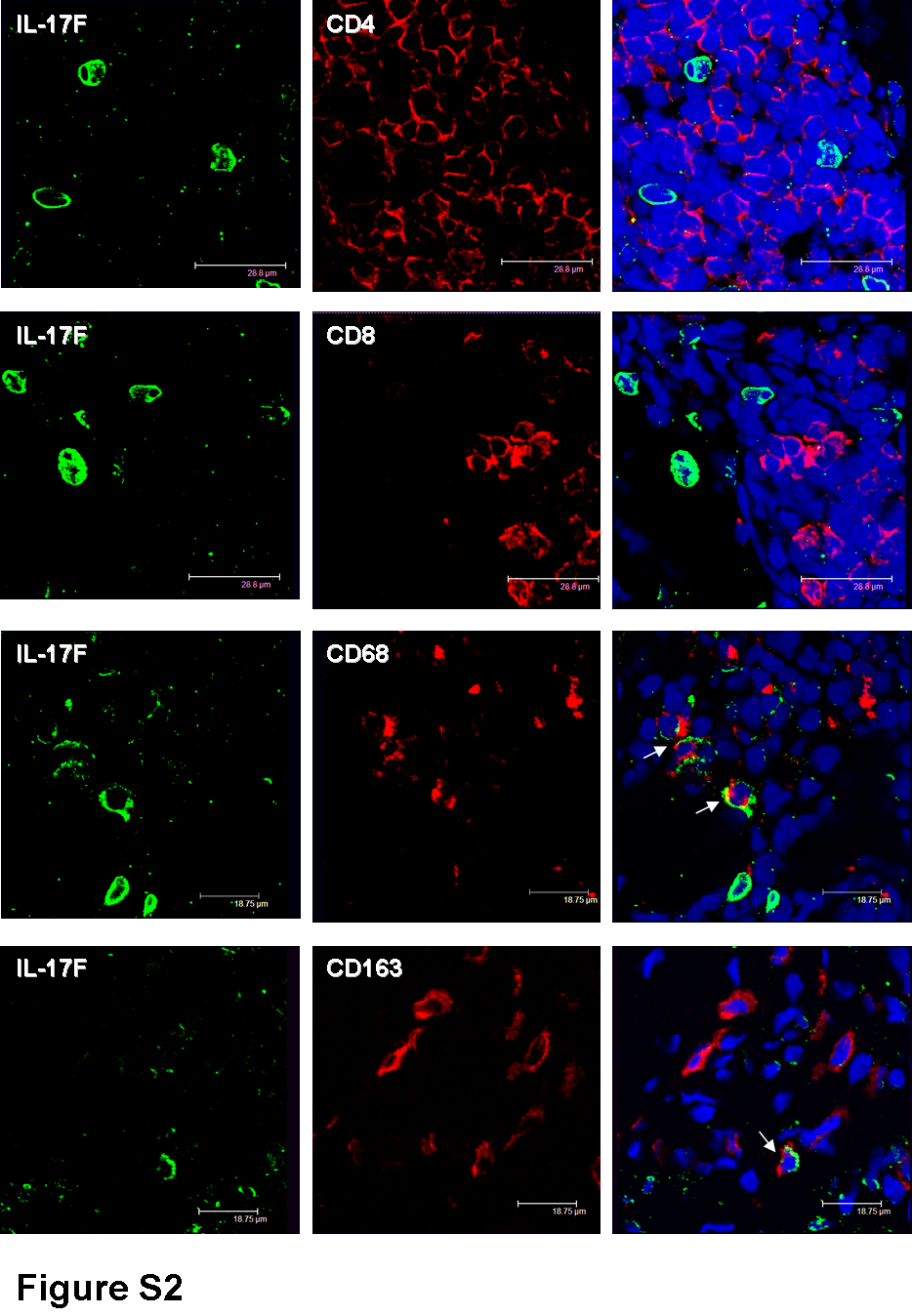

Supplement: Additional file 2: Figure S2. — Colocalisation between IL-17F and CD4, CD8, CD68 and CD163 was examined using double-immunofluorescence labelling and visualized by confocal microscopy. Representative pictures showing separate channels are presented. [file 13075_2014_426_MOESM2_ESM.png]
